# Against the motion: Lasers are superfluous for the surgical management of benign prostatic hyperplasia in the developing world

**DOI:** 10.4103/0970-1591.56188

**Published:** 2009

**Authors:** Anil Varshney, Anshuman Agarwal

**Affiliations:** RG Stone Urology and Laparoscopy Hospital, F 12, East of Kailash, New Delhi - 110 065, India

**Keywords:** Benign prostatic hyperplasia, HoLep, lasers

## Abstract

Lasers have arrived in a big way for the management of benign prostatic hyperplasia. The most common ones in use are holmium, potassium titanyl phosphate (KTP) and thulium. They remove the prostatic adenoma either by way of enucleation or ablation. Backed by numerous studies that prove their safety, efficacy and durability, lasers score over TURP in several ways. Their use is associated with less blood loss, shorter catheter time and decreased hospital stay. The fluid absorption during laser prostatectomy is negligible and thus makes it safer for use in cardiac patients. Also there is no chance of a transurethral resection syndrome, the incidence of which is approximately 2% with TURP. Due to superior hemostatic capabilities and non interference lasers can be used in patients on anti coagulants, cardiac pacemaker. Another advantage of laser over TURP is its ability to deal with prostates that are larger in size especially holmium laser which has been used to enucleate glands more than 300 g in size thus completely avoiding the need for open prostatectomy. The amount of tissue removed with enucleation is more thus retreatment rates are less than that of TURP. The initial cost of laser is higher but its capability to treat stones, its use in high risk situations, less morbidity, short hospital stay, and durable results make it an attractive option to treat BPH even in the developing world.

## INTRODUCTION

Transurethral resection of prostate (TURP) was introduced as a method of treatment that was less invasive than open prostatectomy. It took at least 50 years for it to be accepted as the established treatment and it took even longer for its exact role in the therapeutic armamentarium to be found. But, “it is very likely that someday TURP will be replaced as the ideal interventional treatment for benign prostatic hyperplasia (BPH)”.[1]

Lasers have arrived in a big way for the management of BPH. Their effectiveness and safety is backed by numerous randomized controlled trials (RCTs) and case series.

Three lasers, viz. holmium, potassium titanyl phosphate (KTP) and thulium are in fray of gaining superiority over each other. The technique of laser prostatectomy has evolved over the last decade and now holmium is used mainly for enucleation, KTP for vaporisation and thulium can be used for resection and vaporization.

In the developing world, major issues with lasers are cost, availability and technical know-how. TURP and open prostatectomy are the main competitors of laser. Despite numerous modifications and innovations in the TURP technique and instrumentation, the morbidity and mortality statistics have not changed over the last 15–20 years (Mulligan *et al*. 1997).[2] The developing world, however, implies that things are improving and thus treatment offered for BPH should also improve, and it would be unfair to deprive the people living in the developing world of the advantages of laser in the management of BPH.

Any modality for the treatment of BPH should be evaluated on the following parameters: relief of obstruction, durability, morbidity and safety, versatility and cost effectiveness. Let us examine all these parameters in the light of the currently available literature.

## RELIEF OF OBSTRUCTION

Holmium laser enucleation of the prostate (HoLEP) is an endoscopic equivalent of open prostatectomy, which is considered as a gold standard for the relief of obstruction. It involves complete enucleation of adenoma and subsequent removal of tissue by morcellation. An RCT comparing open prostatectomy with HoLEP has proven its efficacy, with much less morbidity.[3,4] The duration of catheterization and hospitalization was particularly striking as the values for HoLEP were less than half of those for the open procedure (1.5 vs. 4.1 days of catherization; 2.7 vs. 5.4 days of hospitalization).

HoLEP has been shown to be superior to TURP for relief of bladder outlet obstruction as the tissue removed is greater with HoLEP.[5] Gilling reported a statistically significant difference in detrusor pressure at maximal flow 6 months post-operatively between HoLEP (from 76.2 cm of water to 20.8 cm water, a 73% reduction) and TURP (from 85.5 cm water to 40.7 cm water, a 52% reduction) (*P*<0.001).

The results of HoLEP in acute urinary retention were found to be better than that for other surgical modalities probably due to the completeness of adenoma removal as almost all patients with urinary retention treated by HoLEP were able to void post-operatively.[6]

Photoselective vaporization (PVP) using KTP laser results in a TURP-like cavity. Malek *et al*.[7] reported a 75% improvement in symptom scores and a mean increase in flow rates of 250% at 3 months with PVP. None of their patients required irrigation in the post-operative period and the duration of catherization was less than 24 h in all.

## DURABILITY

All patients want a one-time solution to their problem. Whereas TURP remains a very effective treatment, second intervention is necessary in 10–15% of the patients within 10 years.[8] Ablative techniques can be expected to have higher retreatment rates as the amount of tissue removed is much less. In a series by Gilling *et al.*, the retreatment rate after HoLEP was 1.4%, which is superior to TURP.[9] Prostate-specific antigen (PSA) decline is considered as a marker for the amount of tissue removed. An 82–86% fall in PSA is expected after HoLEP, which shows near-complete removal of adenoma.[10] Also, the reduction in transrectal ultrasound (TRUS) volume with HoLEP is 76% at 3 years.[10] However, with a vaporization technique as KTP laser, the PSA decline is 17%.[11]

KTP laser has been used to treat prostates >100 ml with durable results. In a prospective evaluation among 54 men with a mean prostate volume of 135 ml, three patients required repeat PVP for recurrent lower urinary tract symptoms within 18 months and one patient required open prostatectomy at 31 months for hematuria.[12] The improvement in the International prostate symptom score, PVR, quality of life and Qmax was sustained at 24 months of follow-up. The reduction in PSA and TRUS volumes from the baseline was also significant.

## MORBIDITY AND SAFETY

Although regional or general anesthesia is required for TURP, HoLEP or KTP ablation, patients undergoing laser treatment can be treated on outpatient basis/discharged earlier with short catheter times.[11,13] This is unlikely with TURP, because bleeding after the procedure increases the catheter time and thus hospital stay remains a problem with this technique.

Mebust *et al.* retrospectively analyzed 3885 patients undergoing TURP, with a mean of 22 g resected, and found that the risk of blood transfusion was 2.5% and the risk of transurethral resection(TUR) syndrome was 2%.[14] Prostates of > 45 g had a significantly higher incidence of intraoperative bleeding and TUR syndrome. Muzzonigro *et al.* prospectively analyzed a cohort of patients with TRUS-calculated prostate volumes of 70–150 ml who had TURP.[15] These patients had an 8.9% incidence of blood transfusion.

In a randomized prospective study comparing standard TURP, HoLEP and transurethral vapour resection of prostate, Gupta *et al.* reported less amount of blood loss with HoLEP when compared with TURP.[16] Also, the duration of catheterization was least for HoLEP.

The incidence of nursing events after laser surgery for prostate is much less than TURP. HoLEP was found to be safe even in patients with acute urinary retention requiring surgical intervention who were previously reported to be at a greater risk of adverse events.[6]

As lasers are less likely to cause bleeding, Elzayat *et al.*[17] studied the safety and efficacy of HoLEP in a series of 83 patients with bleeding diathesis. Only seven of these patients required blood transfusion and one required platelet transfusion due to bleeding, which coincided with the resumption of anticoagulation therapy. The outcome measures in this subgroup were no different from other reported series on HoLEP.

Another concern in patients undergoing TURP is absorption of irrigation fluid during the procedure leading to electrolyte imbalance.[14] Lasers use saline irrigation, which should eliminate the risk of transurethral resection syndrome with its associated fluid shifts. Shah *et al.*[18] performed a study to define fluid absorption during HoLEP using the breath ethanol technique with normal saline tagged with 1% ethanol. Fluid absorption occurred in approximately 26% of the patients; however, none of the patients developed transurethral resection syndrome. Using the same technique, Barber *et al.*[19] documented the lack of any significant absorption of irrigation fluid (sterile water) during PVP even for large prostates.

A non-randomized prospective trial by Ruszat *et al.*[20] of PVP using KTP laser was found to be safer than TURP in terms of intraoperative bleeding (3% vs. 11%), blood transfusions (0% vs. 5.5%), capsular perforation (0.4% vs. 6.35%) and early post-operative clot retention (0.4% vs. 3.9%).Thus, they were able to treat older men with larger prostates safely with KTP laser with same functional outcomes as TURP.

Sandhu *et al.*[21] evaluated the safety of PVP using KTP laser in high-risk patients on anticoagulants. They stopped warfarin 2 days before surgery and resumed it the day after. However, antiplatelets were not discontinued. There was no requirement of blood transfusion and no significant fall in hematocrit. None of the patients developed clot retention in the post-operative period.

In another randomized trial by Xia *et al.*,[22] thulium laser resection of prostate was found to be superior to TURP in terms of catheterization times (45.7 ± 25.8 h vs. 87.4 ± 33.8 h; P<0.0001), hospital stay (115.1 ± 25.5 h vs. 161.1 ± 33.8 h; P<0.0001) and drop in hemoglobin (0.92 0.82 g/dl vs. 1.46 ± 0.65 g/dl; P <0.001), whereas time taken was equivalent in both the groups. Subjective and urodynamic improvement were not significantly different in both the groups. Late complications were also comparable.

The safety profile of lasers permits their use in critically ill patients, e.g. with poor cardiorespiratory, renal function. Retrograde ejaculation is universal after a properly performed laser surgery or TURP.

## VERSATILITY

Bladder outlet obstruction is known to result in formation of vesical calculi, diverticulum. Holmium laser in particular has an edge over other forms of treatment. Using the same equipment, fragmentation of vesical calculi can be performed effectively. With Holmium laser, diverticular neck can also be incised if indicated.

Lasers can be used for all sizes of prostates. The vaporization techniques are however more useful for smaller prostates. Matlaga *et al.*[23] reported a series of 86 men with pre-operative TRUS volumes of >125cc who underwent HoLEP. The tissue retrieval rate was 1.09 g/min whereas serum PSA decline was 90.2% and peak urinary flow rate went from a baseline of 9.1 ml/s to 24.9 ml/s at 12 months. These findings are superior to those previously reported for both open suprapubic prostatectomy as well as TURP for patients with extremely large prostates.

## COST EFFECTIVENESS

Initial cost of laser equipment is more than the conventional TURP or open surgery instruments. However, reusability of the laser fibers, durability of the equipment and its use by multiple specialities leads to a significant reduction in the running cost. Multiple soft tissue applications of lasers, e.g. resection of bladder tumor, incision of urethral stricture and ureteropelvic junction, use during flexible ureterorenoscopy and fragmentation of calculi (Holmium laser), help in increasing the scope of their use and thus decrease the cost.

The advantages of laser treatment, viz. less hospital stay, early resumption of activity, less retreatment rates, safety in high-risk patients and capability to treat large prostates, also overcome the cost factor.

Even in the developing world, a group of patients exists who need advanced technology to manage their problem. Also, one-time surgical management for a poor patient with bladder outlet obstruction can turn out to be cheaper than life-long medication with uncertain outcome.

## CONCLUSION

Laser prostatectomy procedures are being embraced with enthusiasm by many surgeons concerned about the morbidity and mortality associated with TURP.

The key safety issue with laser prostatectomy techniques is the amount of bleeding that occurs during and after the procedure. It is the relative bloodlessness that is considered to be one of the primary benefits in comparison with TURP. Completeness of adenoma removal for any size of prostate provides unmatched superiority of lasers, especially HoLEP, in the relief of obstruction and long-term durability.

Laser versatility, reusability of fibers and multispecialty use make it cost effective. Because of their excellent efficacy and minimal morbidity, lasers today occupy a definite place in the surgical management of BPH and cannot be regarded as superfluous even in the developing world.
